# Supplementary material for: Guanylation Reactions for the Rational Design of Cancer Therapeutic Agents
Source: Int J Mol Sci. 2023 Sep 7;24(18):13820. doi: 10.3390/ijms241813820 (PMC10530677; doi:10.3390/ijms241813820)
Supplement: Supplementary file 1 [file ijms-24-13820-s001.zip › ijms-2590520-supplementary.pdf]

SUPPORTING INFORMATION
FOR

Guanylation reactions for the rational design of cancer therapeutic agents.

Almudena del Campo-Balguerías,[†,‡] Blanca Parra-Cadenas,[&] Cristina Nieto-Jimenez,[δ] Iván Bravo,[†,§] Elisa Poyatos,[#] Pavel Gancarski,[#] Fernando Carrillo-Hermosilla,[†] Carlos Alonso-Moreno,[†,‡,] and Alberto Ocaña[δ,]**

[†]Unidad nanoDrug, Facultad de Farmacia, Universidad de Castilla-La Mancha, 02008 Albacete, Spain.

[‡]Departamento Química Inorgánica, Orgánica y Bioquímica. ORCATS group. Facultad de Farmacia de Albacete-Centro de Innovación en Química Avanzada (ORFEO-CINQA), Universidad de Castilla-La Mancha, 02008 Albacete, Spain

[&]Departamento de Química Inorgánica, Orgánica y Bioquímica. COMCAT group. Centro de Innovación en Química Avanzada (ORFEO−CINQA), Universidad de Castilla-La Mancha, E-13071 Ciudad Real, Spain.

[δ]Experimental Therapeutics Unit, Hospital Clínico San Carlos, IdISSC, Fundación Jiménez Díaz, START. 28040 Madrid, Spain.

[§]Departamento Química-Física. Facultad de Farmacia de Albacete, FOTOAIR group, Universidad de Castilla-La Mancha, 02008 Albacete, Spain

[#]Cancerappy, Avda Ribera De Axpe, 28, 48950 Erandio, Bizkaia, Spain.

^{1}H-NMR of *ACB7* in $CDCl_3$.

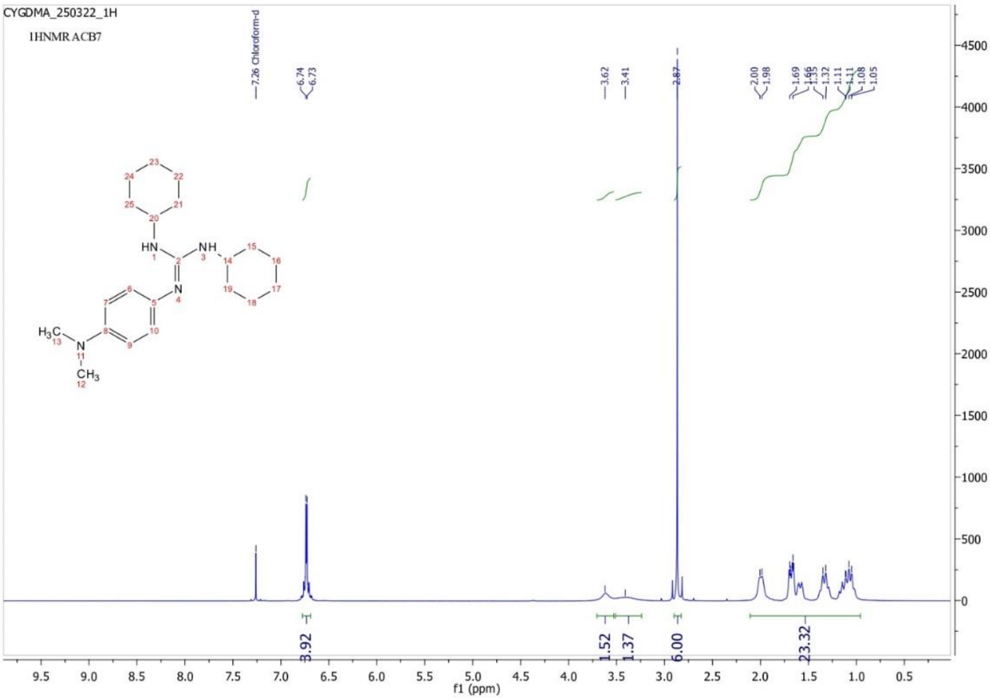

^{13}C-NMR of *ACB7* in $CDCl_3$.

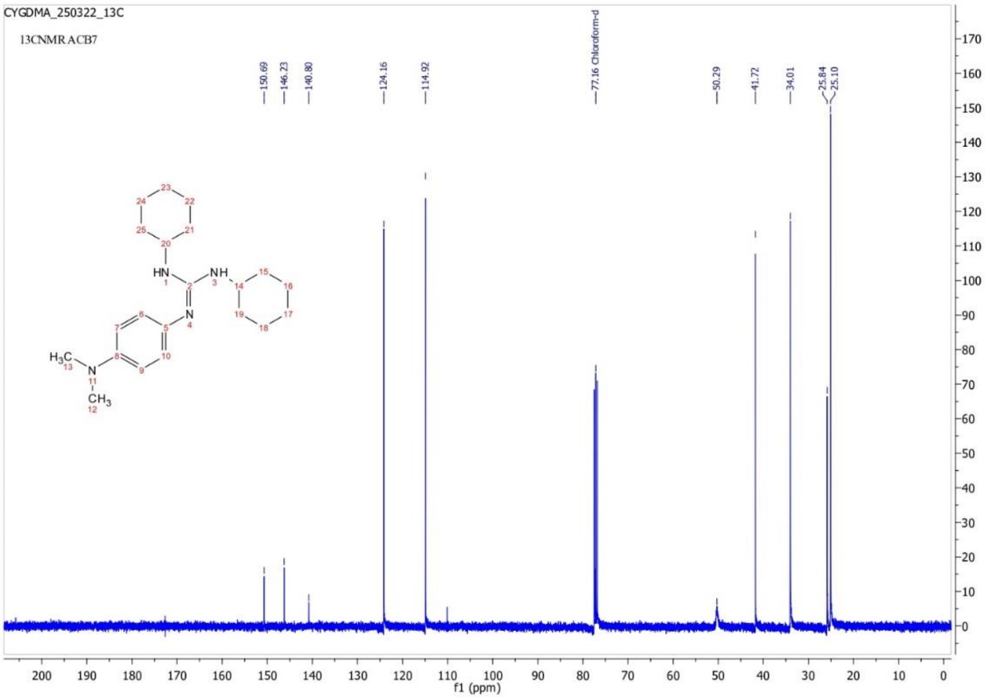

Multinuclear 2D (^1H-^{13}C HSQC) of *ACB7*

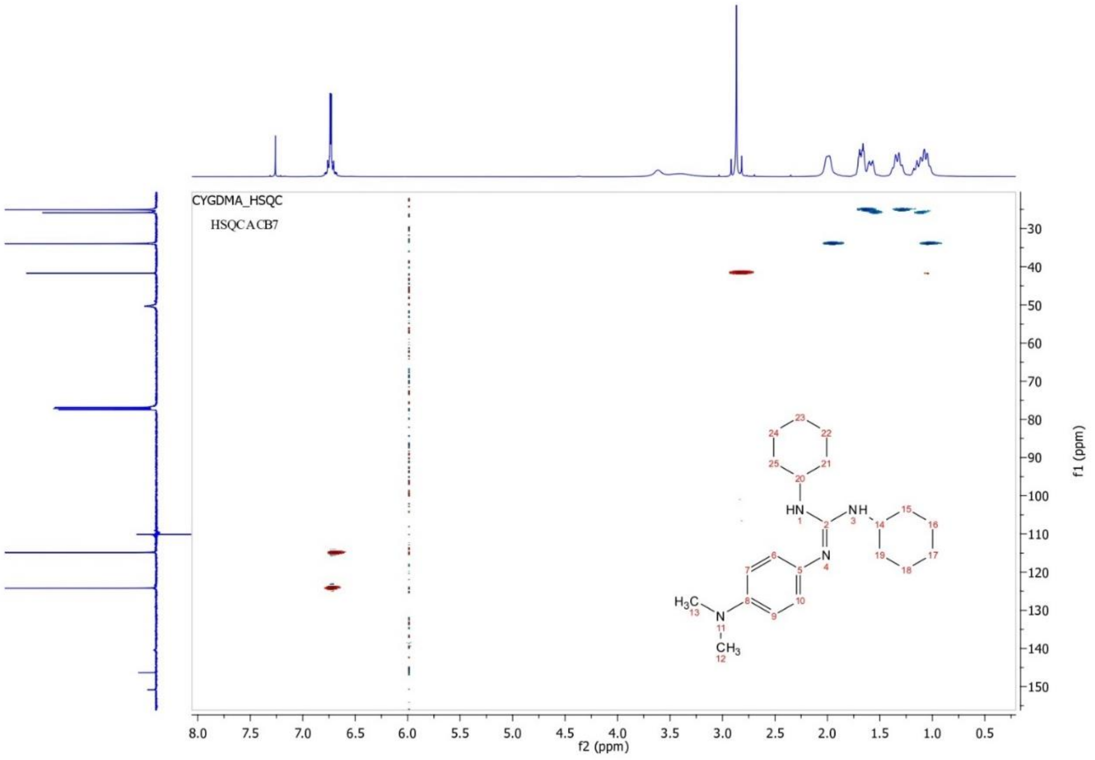

Multinuclear 2D (^1H-^1H COSY) of *ACB7*

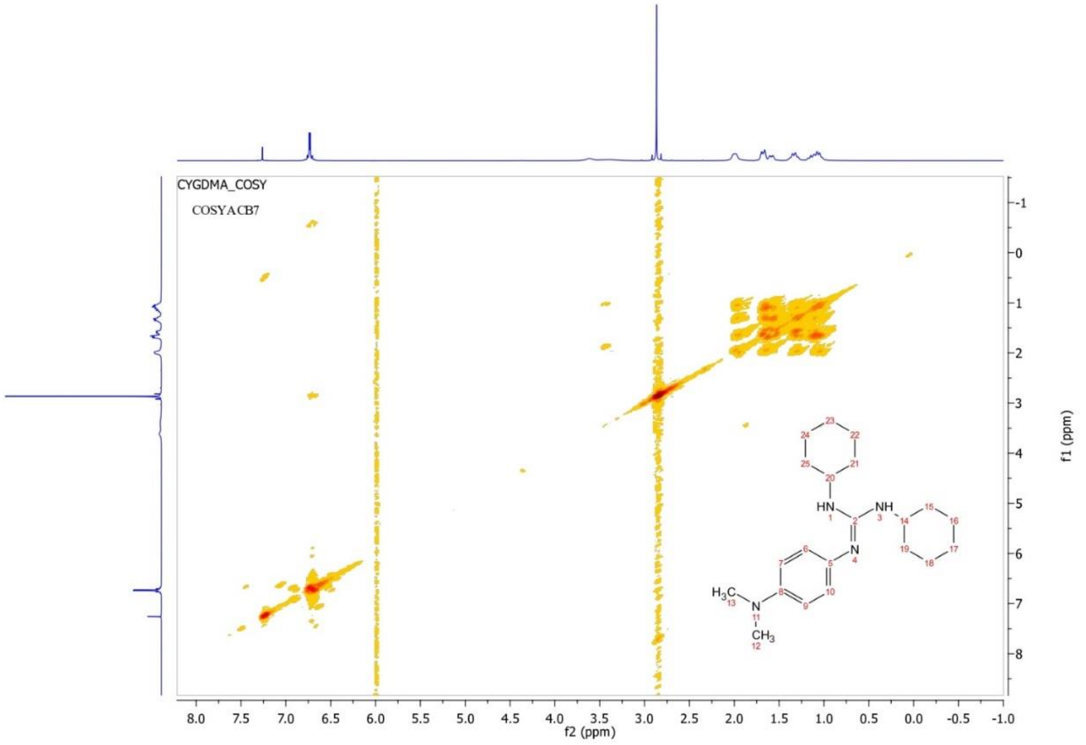

Figure S1. ^1H-NMR, ^{13}C-NMR, multinuclear 2D (^1H-^{13}C HSQC) and multinuclear 2D (^1H-^1H COSY) NMR spectra of *ACB7*

^{1}H-NMR of *ACB10* in $CDCl_3$.

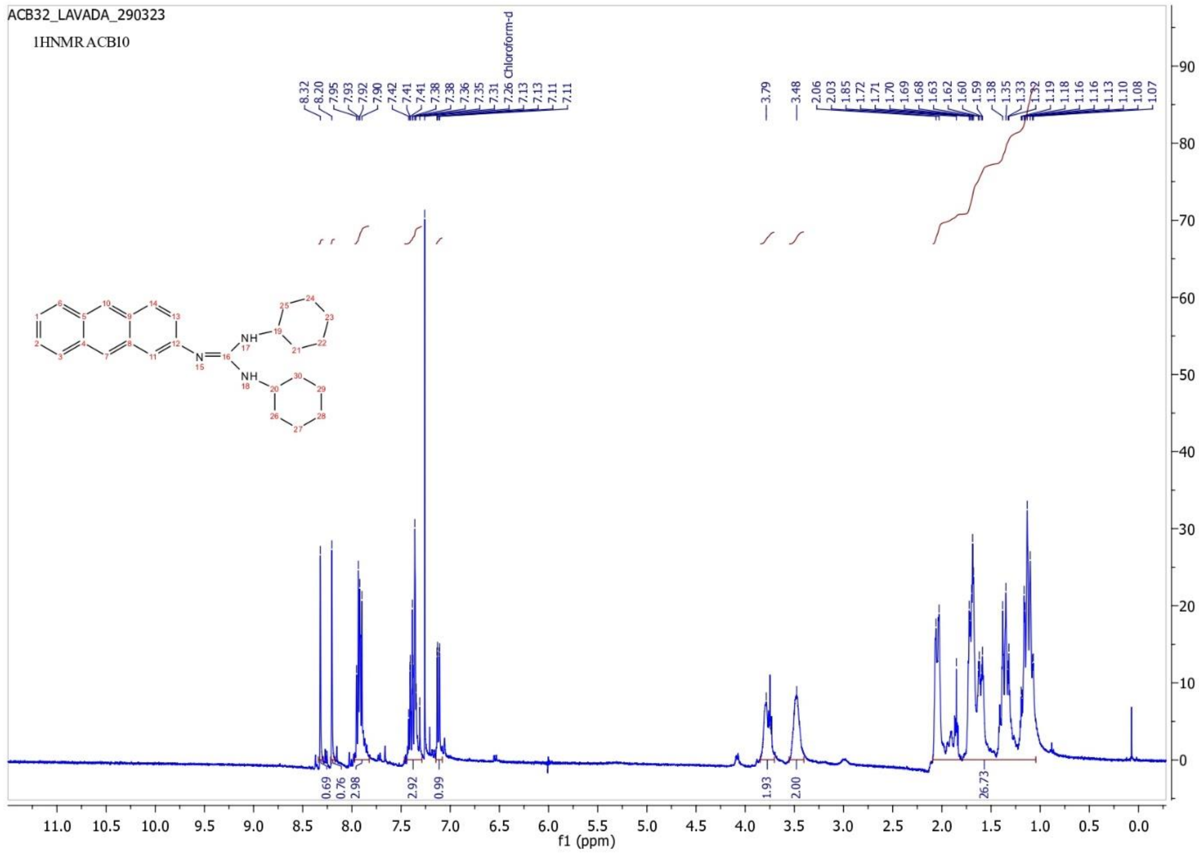

^{13}C-NMR of *ACB10* in $CDCl_3$

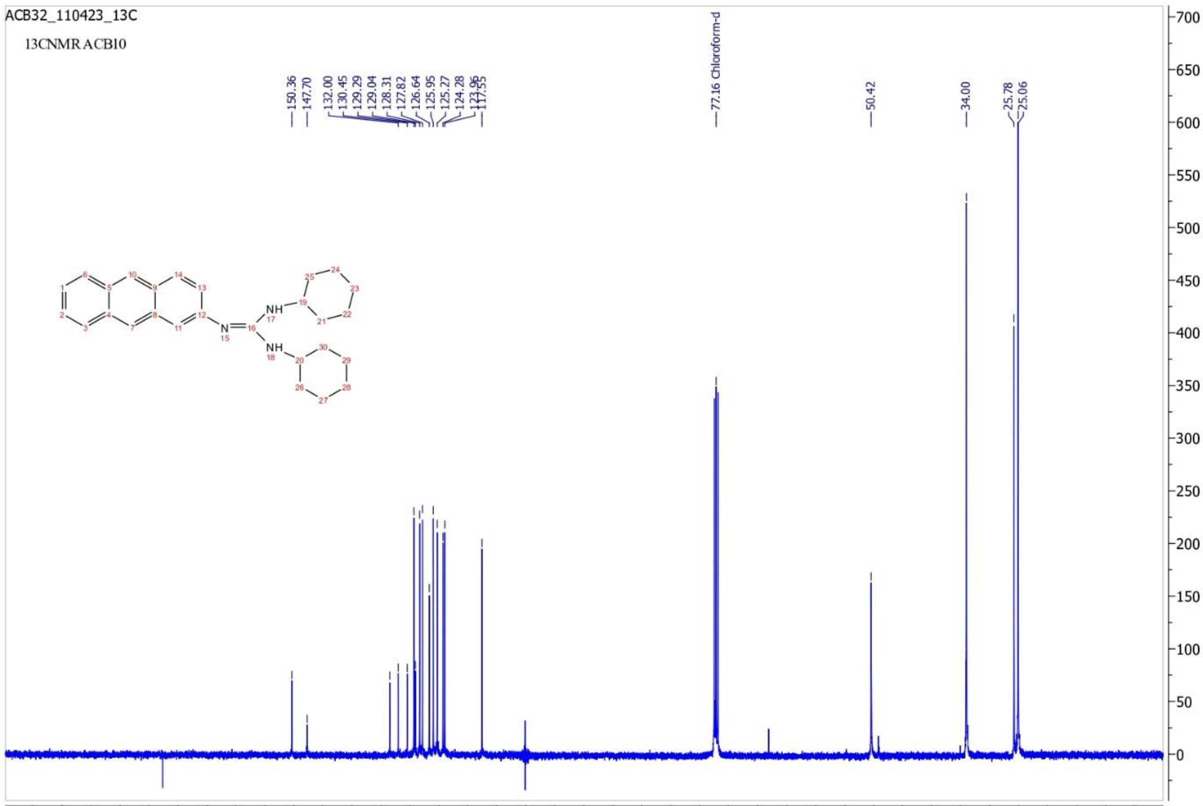

Multinuclear 2D (^1H-^{13}C HSQC) of *ACB10*.

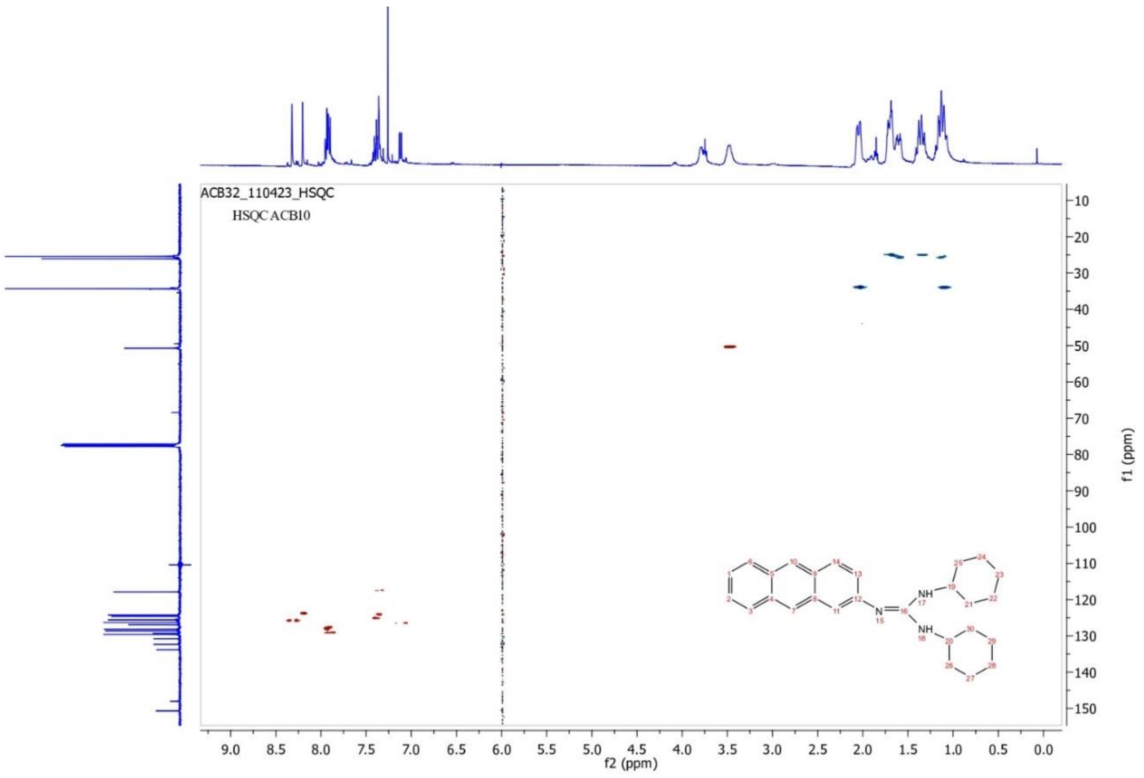

Multinuclear 2D (^1H-^1H COSY) of *ACB10*

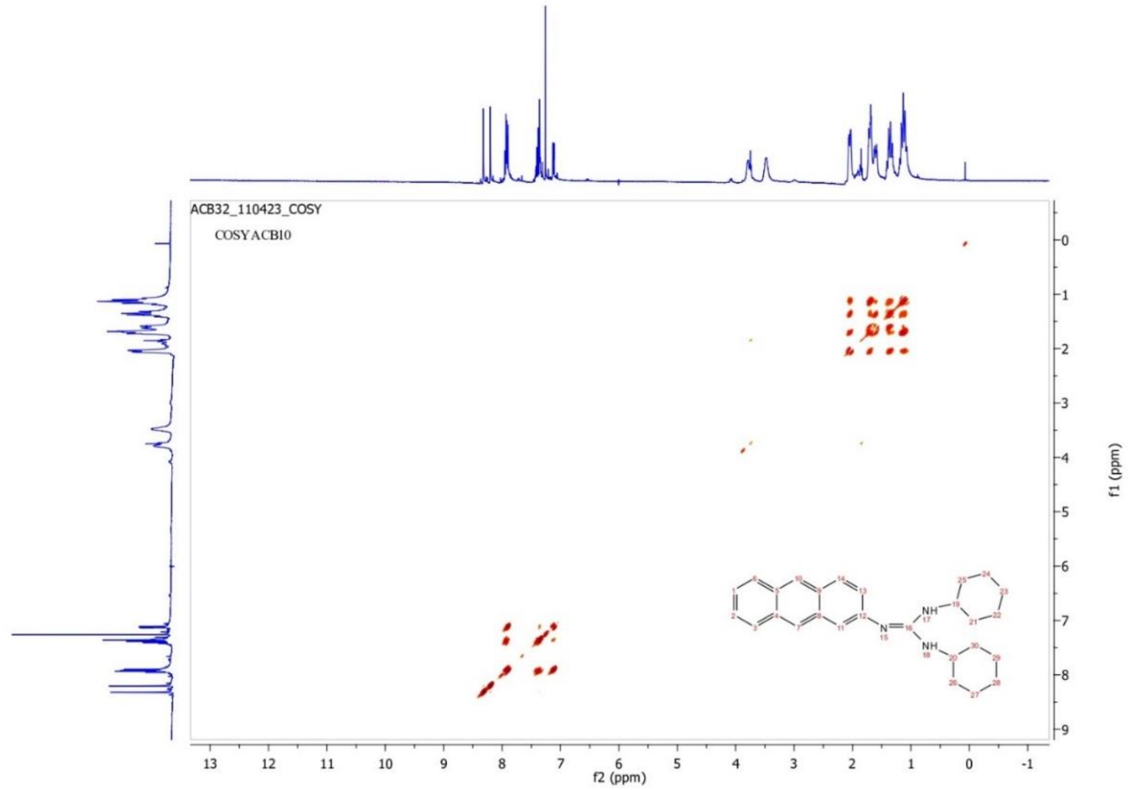

Figure S2. ^1H-NMR, ^{13}C-NMR, multinuclear 2D (^1H-^{13}C HSQC) and multinuclear 2D (^1H-^1H COSY) NMR spectra of *ACB10*.

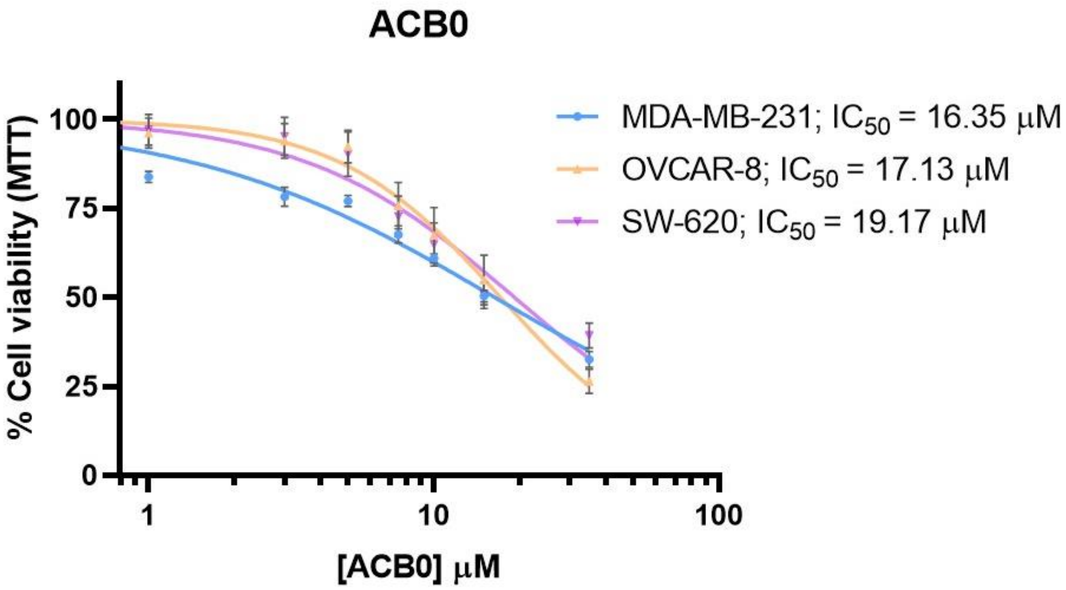

Figure S3. Antiproliferative effect induced by different concentrations of *ACB0* in the indicated cell lines at 72 h tested by MTT assay. Calculation of the concentrations IC50 based on a nonlinear regression curve fit (log(inhibitor) vs. normalized response-variable slope). The mean of three independent experiments (each in triplicate) is plotted together with the standard error (SEM).

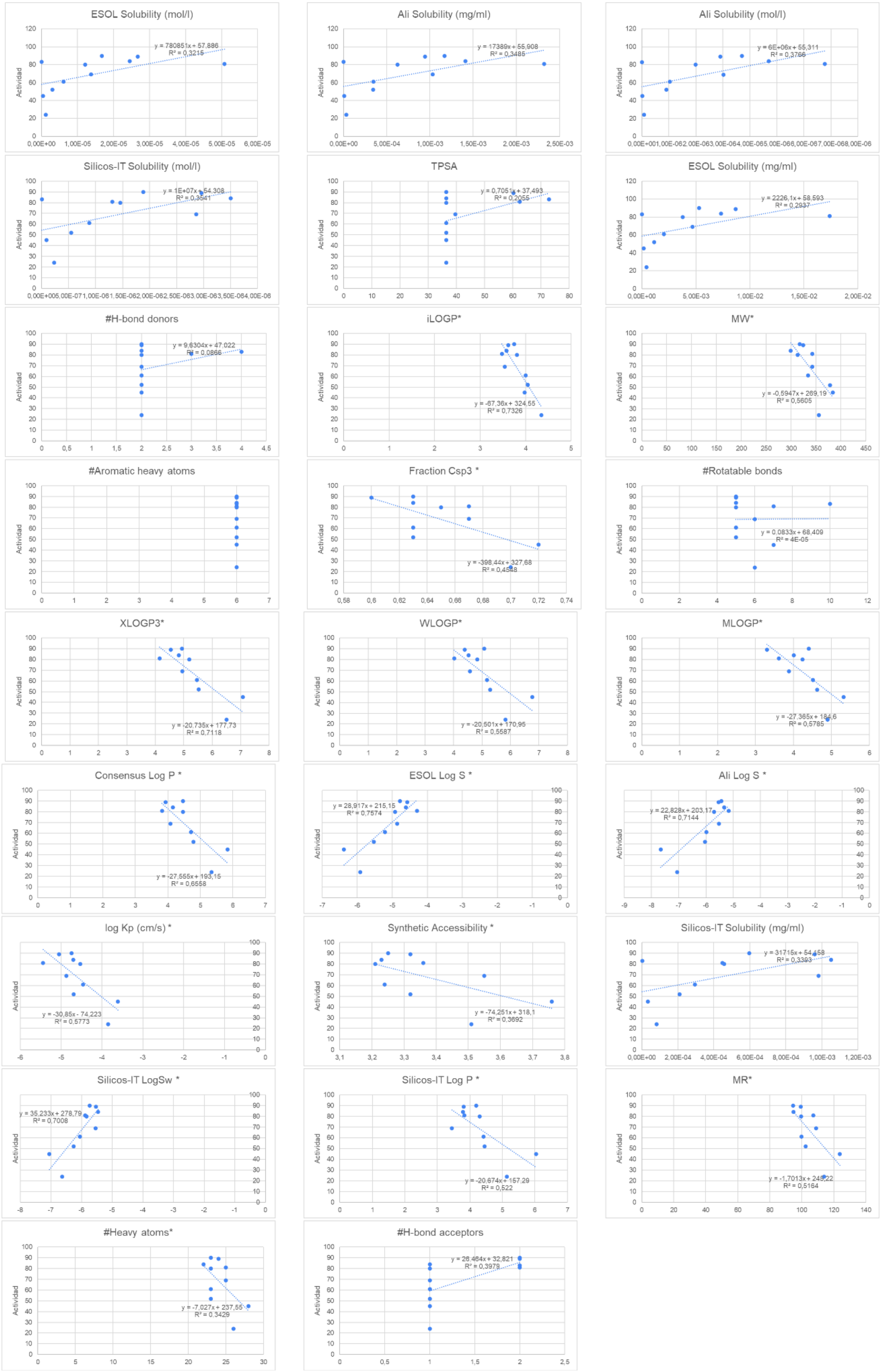

Figure S4: Correlations between ADME parameters.

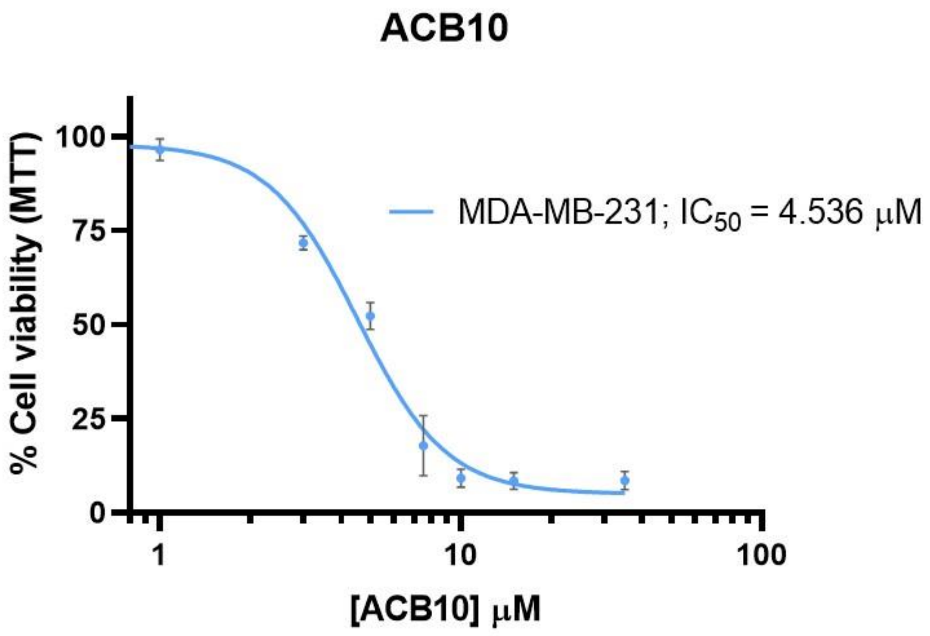

Figure S5: Antiproliferative effect induced by different concentrations of *ACB10* fluorescent guanidine in the MDA-MB-231 cell line at 72 h tested by MTT assay. The inhibitory concentration 50 (IC50) for MDA-MB-231 cell line is shown. Calculation of the concentrations IC50 based on a nonlinear regression curve fit (log(inhibitor) vs. response-variable slope). The mean of three independent experiments (each in triplicate) is plotted together with the standard error (SEM).
